# A novel method for assessing microplastic effect in suspension through mixing test and reference materials

**DOI:** 10.1038/s41598-019-47160-1

**Published:** 2019-07-23

**Authors:** Zandra Gerdes, Markus Hermann, Martin Ogonowski, Elena Gorokhova

**Affiliations:** Department of Environmental Science and Analytical Chemistry, Stockholm University, Svante Arrhenius väg 8, SE-11418 Stockholm, Sweden

**Keywords:** Natural hazards, Environmental impact

## Abstract

The occurrence of microplastic in the environment is of global concern. However, the microplastic hazard assessment is hampered by a lack of adequate ecotoxicological methods because of conceptual and practical problems with particle exposure. In the environment, suspended solids (e.g., clay and cellulose) in the same size range as microplastic, are ubiquitous. Therefore, it must be established whether the addition of microplastic to these background levels of particulate material represents a hazard. We present a novel approach employing a serial dilution of microplastic and reference particles, in mixtures, which allows disentangling the effect of the microplastic from that of the other particulates. We demonstrate the applicability of the method using an immobilization test with *Daphnia magna* exposed to polyethylene terephthalate (test microplastic; median particle diameter ~5 µm) and kaolin clay (reference material; ~3 µm). In the range of the suspended solids test concentrations (0–10 000 mg L^−1^), with microplastic contributing 0–100% of total mass, the LC_50_ values for the plastic mixtures were significantly lower compared to the kaolin exposure. Hence, the exposure to polyethylene terephthalate was more harmful to the daphnids than to the reference material alone. The estimated threshold for the relative contribution of the test microplastic to suspended matter above which significantly higher mortality was observed was 2.4% at 32 mg of the solids L^−1^. This approach has a potential for standardization of ecotoxicological testing of particulates, including microplastic.

## Introduction

The increasing pollution with plastic waste is of global concern. What is more, all plastic debris eventually breaks down to small fragments collectively termed microplastics that are omnipresent in aquatic environments, including alpine lakes, rivers, oceans, and arctic ice^1–4^. The plastic littering is expected to increase because of increased production and continuous discharge^5^. Therefore, research on the environmental assessment of polymer particulates is in high demand. Despite the high public concern, scientists disagree on the immediacy of the MP pollution problem^6–9^, and it remains unclear whether microplastic is harmful to biota, and what are the impact mechanisms. The continuing uncertainty is, at least partly, related to the fact that microplastic is a new type of environmental contaminant with yet unsettled methodology for hazard testing.

The detrimental effects of suspended solids on aquatic biota occur not only with microplastics but also with other particulate material, and both mineral^10–13^ and microplastic^14–17^ particles can alter feeding, growth, and survivorship. Natural processes, such as wind and sediment resuspension, can increase the quantity of nutritionally inert particles in the water; however, human activities, like, dredging and stormwater runoff, may also elevate their concentrations. High concentrations of total suspended solids (TSS) have been found to reduce primary production^18^, suppress zooplankton populations^19^ and alter feeding behavior in fish^20^. Therefore, to protect wildlife, water quality standards are implemented for TSS concentrations or allowable TSS levels in, e.g., stormwater effluents^21^, lakes, and streams^22^.

Regulatory efforts to set allowable microplastic levels are calling for adequate methodological approaches for hazard assessment of particulates, relevant test species, and exposure scenarios. A step towards quantifying hazardous properties of synthetic polymer particles is to develop and apply standardized practices and experimental designs that can provide threshold values of these effects. However, given the presence of various particulates and the hazardous effects of suspended solids at high concentrations, such designs should include not only the microplastic in question but also some environmentally relevant reference material(s). Particular attention should be paid to the similarity of physical properties that are important for biological responses, e.g., size distribution, shape, surface charge, hydrophobicity, etc., between the reference particles and the microplastic^14,23^. Also, to maintain the experimental reproducibility and stable encounter rates in a pelagic exposure scenario, it is important to keep the particulates in suspension during the incubation.

A recent comparison of the effects exerted by microplastic and mineral particulates suggests high similarity in responses across different levels of biological organization, albeit with an indication of a greater hazard by the microplastic^24^. Since natural particles are much more abundant than synthetic polymers in aquatic environments^7^, the hazardous levels of microplastic should rather be presented as a relative contribution of these materials to the suspended solids and not as the absolute concentrations.

To date, there is no standard approach for microplastic effect assessment^25^, despite a rapidly rising number of reports on the effects observed under laboratory conditions. This is partly because it is challenging to design exposure experiments with environmentally relevant concentrations of microplastic based on the commonly reported levels (<10 particles m^3^)^26–28^. Moreover, the existing approaches do not explicitly test the effects of MP *per se* but those of non-caloric particulates in the exposure system. To move the field of MP ecotoxicology forward, we need to develop test methods that (1) are appropriate for delineating effects of different particulate materials in mixtures, (2) provide estimation of the critical concentrations of microplastic in different environments, (3) allow high-throughput testing, and (4) support read-across and categorical assessment of solid polymer particles. Here, we propose a new approach employing a linear dilution of microplastic and reference particles in mixtures, which allows identifying microplastic toxicity while controlling for the total concentration of suspended matter in the experimental system. Further, we demonstrate the applicability of this approach using the 96-h exposure of the cladoceran *Daphnia magna* to a mixture of polyethylene terephthalate (PET) as a test microplastic and kaolin, a soft clay, as reference material.

## Method

**Figure 1:**
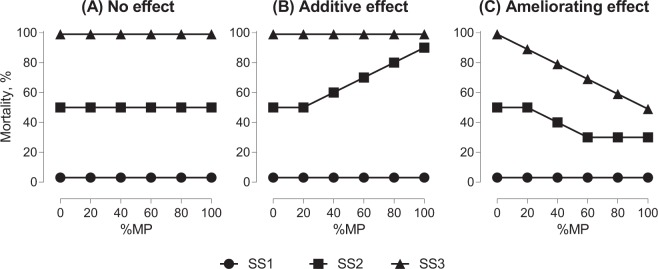
Possible outcomes of the MP Ratio Test shown as a response (e.g., mortality) to the relative microplastic contribution (%MP) to the suspended solids (SS) in the test system consisting of three test concentrations (SS1–SS3) with increasing SS levels. *Scenario* (**A**) No microplastic effect, mortality is responding to the increasing SS level. *Scenario* (**B**) An additive effect of microplastic, above critical mortality threshold, is positively responding to %MP. *Scenario* (**C**) An ameliorating effect of %MP on SS toxicity. Based on existing reports for SS effects on *Daphnia*, no effects are expected at low SS concentrations.

The MP Ratio Test was designed to examine whether a particulate material (test particle) is harmful when co-occurring in a mixture with naturally present particulates (reference particle) across a range of suspended solid concentrations. The rationale is as follows: if the test particle is more harmful than the reference particle, then decreasing its contribution to a mixture with the reference particles should decrease the overall toxicity, assuming additivity of the effects (Fig. 1). When the test and the reference particles are provided at varying proportions for each test concentration of the suspended solids, then, a dose-response relationship can be established for each mixture over a range of the concentrations.

### Test organism

The freshwater cladoceran *Daphnia magna* was used as the test species. These microcrustaceans are the most common model organisms in aquatic ecotoxicology and have been extensively used in studies assessing both mineral particle^14,19,29–31^ and microplastic^14,30,32^ effects. All experimental animals originated from the same clone (Clone 5; The Federal Environment Agency, Berlin, Germany) cultured in M7 media at a density of ~10 ind. L^−1^ and fed a mixture of algae (*Pseudokirchneriella subcapitata* and *Scenedesmus spicatus*) *ad libitum*.

### The reference and test particles

Kaolin (Sigma-Aldrich, K7375) was used as the reference material. It contains mainly the clay mineral kaolinite, a hydrous aluminosilicate that occurs globally in suspended particulates and has been used with daphnids, both as a reference particle when assessing microplastic effects^14^ and as a test particle when assessing effects of TSS^29^. As the test microplastic, we used polyethylene terephthalate (PET, Goodfellow GmbH, product number ES306312; Text 1, Fig. S1, Supplementary Information), to represent a plastic commonly found in the environment. The PET was obtained as 3–5 mm-sized pellets from the manufacturer and milled to a powder by Messer group GmbH, Germany. The powder was first mixed with milliQ water containing 0.01% v/v of a non-ionic surfactant (Tween-80, Sigma-Aldrich), and passed through a 40-µm sieve to produce a size fraction similar to that of kaolin. Both PET (median size ~5 µm) and kaolin (~3 µm) particles showed a unimodal size distribution, with only a few percents of the particles >10 µm and ~90% in the 3–10 µm range; see Text 2 and Figs S2 and S3, Supporting Information, for particle size distribution and description of the particle preparation.

### Test suspensions

Particle stocks were prepared by suspending weighed kaolin and MP in M7 media (reconstituted lake water);^33^ the volumes were adjusted to produce mass-based concentrations of 0.1, 1, 10, 100, 1000, and 10 000 mg L^−1^. To minimize particle aggregation during the preparation of the test mixtures, the stock suspensions were sonicated for 10 min and examined using a light microscope (Fig. S4) before use. The test mixtures based on nominal concentrations were prepared in batches with 0%, 20%, 40%, 60%, 80% and 100% of microplastic contribution to suspended solids; each replicate was treated separately. Each test suspension was then transferred to a 50-mL polypropylene centrifuge tube and used in the exposure system.

### Experimental setup and procedures

Altogether, 24 treatment combinations (%MP × SS concentration; Table S1, Supporting Information) were used; the exposures were conducted in several runs. The number of replicates varied among the treatment combinations; four to six replicates were used for 20%, 40%, 60% and 80% MP, whereas five to 43 replicates were used in the treatments with 0% and 100% MP. Also, three to five particle-free controls were added per run. In addition to the controls, 10 to 1000 mg L^−1^ treatments with 0% MP were included in the experiments repeatedly to confirm consistency of the response. Four SS concentrations were tested for the 20–80% MP treatment combinations and six for 0% and 100% MP.

**Figure 2:**
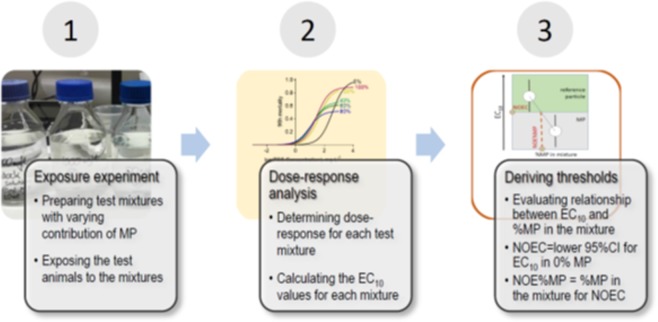
The workflow for deriving threshold values by MP Ratio Test includes (1) exposure experiment with mixtures of known suspended solid concentrations and varying proportions of the test particles (e.g., microplastics, %MP), (2) analysis of the dose-response relationships and calculating EC_10_ values for each mixture, and (3) deriving the NOEC for suspended solid concentration and NOE%MP, which is the percentage of microplastic corresponding to NOEC in the mixed solids.

The outline of the MP Ratio Test and data evaluation procedure included three steps: exposure experiment, dose-response analysis, and calculation of the threshold values (Fig. 2). To do the exposure, we conducted the *Daphnia* sp. acute immobilization test (OECD 202), with some modifications. The standard *Daphnia* immobilization test assesses 48-h mortality (immobilization) in individuals exposed to a range of test concentrations. In our pilot experiments with kaolin, only <20% mortality over 72 h were observed, which was in agreement with low effects on *D. magna* mortality exerted by nano-^34^ and microplastic^35^. In line with these studies, to achieve a higher mortality, we prolonged the test duration to 96 h. Ten neonates (<24-h) were placed in each 50-mL test tube with the exposure media, including the controls. When sealing the test tubes, care was taken to avoid trapping air bubbles inside the tube. The tubes were mounted on a plankton wheel (Fig. S5) with a rotation speed of 0.5 rpm in a thermo-constant room at 21 °C with a light: dark cycle of 16:8 h; at this rotation speed, no sedimentation was observed. The test was terminated after 96 h by counting live and dead daphnids and calculating proportion of immobilized animals for each replicate. The daphnids not able to swim within 30 seconds after gentle agitation of the test medium were considered immobilized (dead).

### Statistical analyses

For each mixture, the LC_50_ values and the corresponding 95-% confidence intervals were calculated using replicate-specific mortality values as observations and a dose-response curve fitting with three-parameter logistic regression (GraphPad Prism, v. 7.0; GraphPad Software, La Jolla California USA). The Abbott’s correction was used to account for the particle-free control mortality (≤10% in all cases)^36^. To generate comparable dose-response curves, 0 was used as the bottom constraint and 1 (i.e., 100% mortality) as the top constraint (Fig. 2, Panel 2). Moreover, only SS concentrations that were common for all treatment combinations were used. To calculate LC_50_ for 0% PET, we used balanced replicated subsampling without replacement from the entire dataset (Table S1) which decreased the number of replicates to five and thus approach the same number of observations as in other treatments. The LC_50_ values were compared across the treatments to evaluate the effect of %MP in the mixture; the non-overlapping confidence intervals were used as evidence of a significant difference between the treatments. Further, the corresponding LC_10_ values were calculated for each treatment and one-phase exponential decay function was fitted to describe the relationship between the LC_10_ values and %MP. As a surrogate for the no-observed effect concentration (NOEC) with regard to suspended matter (SS), the lower bound of its LC_10_ 95%-confidence interval^37^ for 0% MP was used. The critical threshold for %MP present in the mixture and exerting no effect in the test system was termed NOE%MP (No Effect Percentage of Microplastics; Fig. 2, Panel 3).

## Results

**Figure 3:**
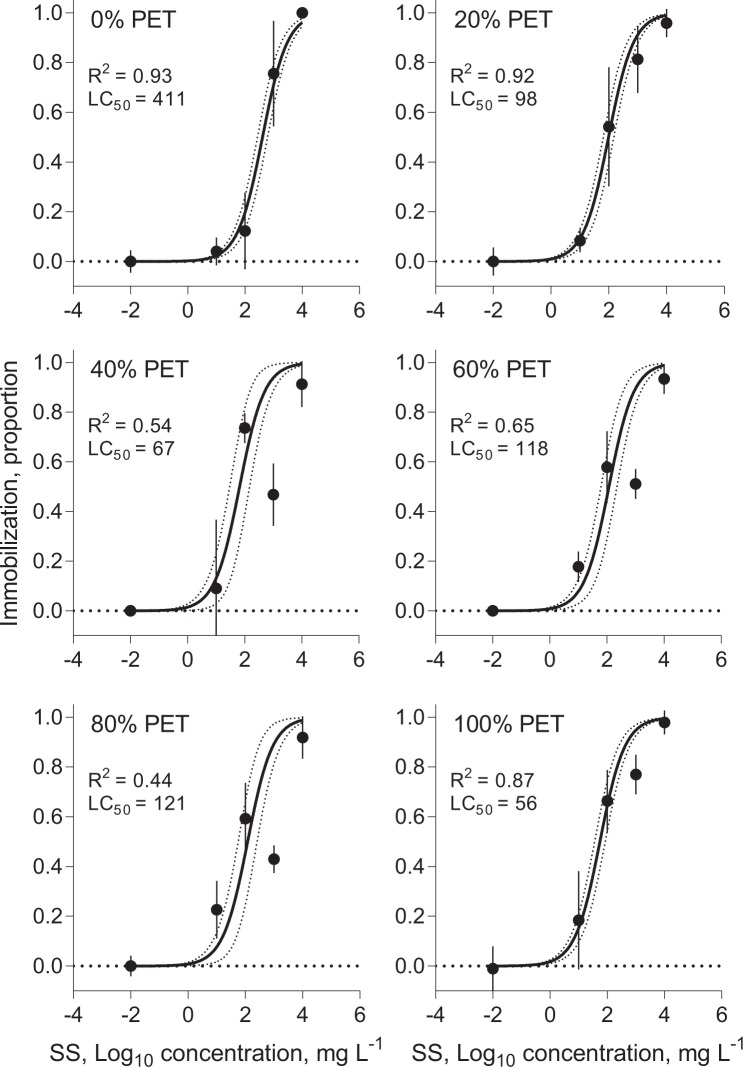
The dose-response curves for *Daphnia magna* exposed to the tested mixtures of PET (0% to 100%) and kaolin during 96 h. The concentration of the suspended solids (SS, mg L^−1^) is based on the mass-based concentration of the total particulate material in the mixture. The data are shown as mean and SE, and the dotted line around the curve indicates 95% confidence interval; the number of replicates per treatment is 5 to 6. For each mixture, the estimated 96-h LC_50_ values (mg L^−1^) and R^2^ of the curve fit are shown.

**Figure 4:**
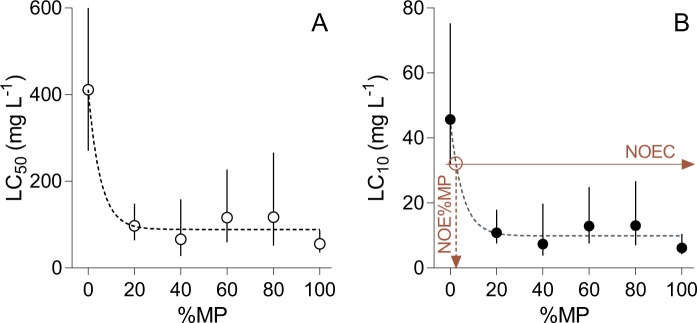
The estimated LC_50_ (**A**) and LC_10_ (**B**) values (mean and 95% confidence interval) for *D. magna* as a function of the mass contribution of MP (%MP, 0% to 100%) to the test mixtures comprised by MP and kaolin. One-phase exponential decay was used to find the LC_10_ for the MP-kaolin mixture corresponding to the lower bound of the confidence interval (shown as intersection point on the B panel) for the kaolin treatment (NOEC; left *y*-axis) and the no effect level of %MP in the test system (NOE%MP; *x*-axis).

Survival in the particle-free controls was consistently high (average 95.4%), and mortality in relation to suspended solids followed the expected concentration-dependent response (Fig. 3; Table S2, Supporting Information). The LC_50_ values in the treatments with 20% to 100% PET were lower than in the 0% PET treatment, with mean values of 56–121 mg L^−1^ and 411 mg L^−1^, respectively (Figs 3 and 4); moreover, the deviation was significant as indicated by the non-overlapping confidence intervals in 0% PET compared to the plastic-containing treatments (Fig. 4A). The highest mortality was observed in the 100% PET treatment.

The one-phase exponential decay function provided an adequate fit for the relationship between the LC values and the %MP in the mixture (LC_50_: R^2^ = 0.95 and LC_10_: R^2^ = 0.99). The fit for the LC_10_ values was used to derive the %MP threshold (NOE%MP) above which significantly higher mortality was observed. This value was determined as 2.4% of PET in the mixture with 32 mg L^−1^ SS (Fig. 4B).

## Discussion

### Test performance

Using the MP Ratio Test, we have shown that addition of microplastics to natural suspended solids resulted in detrimental effects in *D. magna*. Moreover, the test design allowed estimating critical levels for the suspended microplastic that can be considered as hazardous. The threshold level of the microplastic contribution to suspended solids is an important outcome of our test approach because the effects of particulate contaminants, such as microplastic, depend not only on the absolute concentration but also on their relative contribution to the suspended matter or sediment; the latter was also recently shown by Redondo-Hasselerharm and co-workers^38^.

Ecotoxicological data describing effect thresholds in ecologically meaningful settings are needed in microplastic research to support the hazard assessment of solid polymer particles. The MP Ratio Test can be used as a tool for screening a variety of materials and particle types as well as for selecting suitable test organisms and endpoints. It is conceptually similar to both the already standard bioassay approach for testing toxic effluents and sediments by serial dilutions with a reference sediment^39^ and algal toxicity in mixtures with varying proportion of the test species^40^. Furthermore, the need for well-characterized reference materials when evaluating, for example, particle size effects, is also recognized in nanomaterial toxicity assessment^41,42^. However, to our knowledge, our study is the first to assess the effects of suspended MP in mixtures with natural particles using a dose-response approach.

### PET toxicity estimates

We found PET suspension to be more hazardous than kaolin for *D. magna*. The addition of PET powder to the kaolin increased *Daphnia* mortality, with LC_50_ values dropping more than 4-fold in mixtures with >20% of MP. Moreover, particle mixtures containing more than 32 mg suspended solids L^−1^ with more than 2.4% of PET by mass were predicted to exert significantly higher mortality for daphnids upon exposure compared to the suspended kaolin. Thus, 32 mg L^−1^ would represent NOEC of suspended solids in the tested size range (Fig. 2), whereas 2.4% of PET would represent NOE%MP in seston.

The effect concentrations of MP reported in the literature are highly variable and span orders of magnitude even within the same level of biological organization^24^. Unfortunately, only a few reports provide dose-response data for MP suspensions^30,35,43^, and reference particles are rarely employed^14,30^. Although truly comparable published data on PET toxicity for daphnids are not available, some reports are still relevant. For example, a 6-day static exposure of the copepod *Parvocalanus crassirostris* to 14 mg L^−1^ of PET (<11 µm; assuming that particles are spherical, with a density of 1.38 g cm³) was found to decrease population size^44^. This concentration is more than double of our LC_10_ for 100% MP. The difference in the observed response, at least partially, can be attributed to the fact that our animals were starved during the exposure. Moreover, the suspended amount of PET in the study with *P. crassirostris*^44^ is uncertain, because of the static exposure and the lack of information on the mass of MP in the system. For other planktonic filter-feeders and various MP, the reported LC_50_ values are similar to what we have found for PET using the MP Ratio Test. For example, a 96-h LC_50_ of 57 mg L^−1^ was reported for *D. magna* neonates exposed to 1-µm polyethylene (PE) particles, although some mortality was observed already at 13 mg L^−1 ^^35^. Also, *D. magna* exposure to PE fragments (10–75 µm) produced a 48-h LC_50_ of 65 mg L^−1 ^^43^. These findings are comparable with the 96-h LC_50_ observed in the 100% MP treatment (56 mg L^−1^) as well as in the mixtures (66–117 mg L^−1^).

The variation between the reports could be related to possible variability in MP aggregation and settling during the exposure as well as in specific properties of the polymers and particle size^45^. The differences in the experimental setup highlight the importance of both particle characterization in MP research^25^ and keeping the test particle in suspension when using pelagic feeders. Still, based on either modeled data or some of the highest levels reported in the ocean^7,46^, the levels of MP accessible for zooplankton in nature are much – approximately two to four orders of magnitude – lower than the experimentally determined MP levels for the observed effects.

### Important issues in experimental design

The logistics of our approach make it possible to examine a large number of treatments (e.g., SS concentrations, plastic material, particle size, and shape) with reasonable effort. When testing the PET effects on the daphnid survivorship, one person was able to handle 50 experimental units per day routinely. Further developments should include a higher number of treatments with lower %MP to provide more ecologically meaningful test suspensions and improve the threshold estimates for the hazardous MP levels. Furthermore, when more information of the environmentally realistic exposure levels becomes available, future experimental designs can focus on narrowing concentration ranges, as well as including more sensitive endpoints, such as growth and reproduction, but also biochemical responses. When testing PET, for example, a higher resolution of the %MP at the lower range (<20%) would have provided a more precise estimate of NOEC and the corresponding %MP in the mixture. Also, various reference particles can be used depending on the research context, both natural and anthropogenic.

The selection of the reference particles is not a trivial task. One possible criterion is particle size distribution because size spectra for MP and many naturally occurring particles overlap (clay: <2 μm, silt: 2–50 μm, and sand: 50 μm –2 mm)^41^. Another recently advocated option is to benchmark the test particles to the reference MP using reference plastics supplied by commercial vendors^25^, which have a narrow size range and reliable certificates of the chemical and physical properties. Such reference particles would add credibility to any adverse effects exerted by the material in question. Here, we used kaolin, because it has been relatively well studied and broadly used as a model particle in hazard assessment of suspended solids^47^. Several studies also suggest its low toxicity for daphnids^24,48^, which was supported by our 96-h LC_50_ of 411 mg L^−1^. However, in chronic tests with *D. magna*, Robinson and colleagues observed a 7-d LC_50_ of 74 mg L^−1 ^^29^, which can be related to both delayed effects and possible difference in kaolin composition and aggregation during the test. Kaolin powder is a commercially available standard product, but depending on the vendor, it might contain impurities and vary in particle size distribution.

When testing effects of suspensions on planktonic organisms, it is essential to prevent that particles sediment or float, because it will affect the encounter rate and intake by the animals. Moreover, particles with different specific gravity will settle with different rates; hence performing tests under static conditions would not provide stable exposure levels. The exposure of planktonic organisms should preferably be conducted using a plankton wheel that keeps particles in suspension, thus, ensuring stable exposure conditions^49,50^. In plankton ecology, the use of a plankton wheel is a standard procedure when conducting grazing experiments because it minimizes the sedimentation of algae. Although less common in ecotoxicological testing, the plankton wheel has been used to assess the effects of suspended clay and other particulate materials on planktonic filtrators^51^. Even though this method requires some additional effort compared to static exposures employed in OECD tests for soluble chemicals, it is a necessity for standardizing the exposure conditions for particulate materials.

Another area of concern with respect to the standardization of the test procedures is particle behavior, such as aggregation and settling, during the exposure. In this study, we have not monitored changes in the particle size distribution during the exposure. However, the higher LC_50_ values and, especially, lower mortality at 1000 mg L^−1^ compared to 100 mg L^−1^ in the 40% to 80%-PET treatments (Figs 3 and 4) suggest that at high SS levels and in mixtures particles aggregate, and their interactions with the animals decrease. Using the filter-based TSS measurements or analysis of turbidity would improve the control over the test concentrations and allow relating these concentrations to the standard water quality parameters. Moreover, since suspended particles may have complex behavior, their dispersion stability during the exposure should be controlled using size spectra analysis or turbidity measurements. The extent of the aggregation, i.e., aggregate size, composition and cohesion, is dependent on the complex interplay between various components, such as particle material, size, physicochemical properties of the particles and their concentration, but also media ion composition, and organic material, such as food and faeces, present in the system. Aggregation may have affected the concentrations of both PET and kaolin that daphnids encountered during the exposure, and size spectra analysis could potentially explain the non-linear response of LC to %MP. The relationship between the aggregate formation and the measured response is particularly relevant at high SS concentrations. On the other hand, aggregation, at least to some extent, is preventable by, for example, dispersants and sonication, which may be sufficient in short-term tests. These methods are employed in nanomaterial effect assessments^41^ and, in some cases, microplastic studies^52^.

The exposure duration is yet another issue that needs to be considered in acute tests. Daphnid energy expenditure may increase in the presence of non-food particles since the induced filtering activity is similar as for food particles^53^, while the cost of cleaning appendages and egestion through postabdominal rejections may increase^10^. Also, sensitivity to the non-caloric material would increase with starvation, and the higher energy expenditure may decrease survival. In *D. magna* neonates, the critical exposure time is 96 h, which marks the depletion of their fat reserves^54^. Earlier studies using the *D. magna* acute immobilization test for particle suspensions have also shown that it is suitable to extend the exposure period to 96 h from the standard 48 h to increase the sensitivity of the test^35^. However, if a test organism other than *Daphnia* is used, the duration of the exposure must be adjusted depending on its capacity to withstand starvation.

### Implications for regulatory measures and concluding remarks

Many suspension- and filter-feeders frequently face turbid environments with high concentrations of non-caloric materials generated by natural processes, such as terrestrial runoff, currents, and weather-induced bottom sediment resuspension^55^, but also by anthropogenic activities, such as dredging and capping of contaminated sediments using various technologies. In aquatic ecology, conditions with elevated suspended solids are commonly acknowledged as stressful^56^ and regulated by water quality standards^21,57^. The quality standards vary across regions and types of aquatic systems, e.g., lotic and lentic, as well as systems with different natural levels of suspended solids. For example, the Alaskan state standard for clear-water lakes is a maximum increase of TSS, above background levels, equivalent to 25 mg L^−1^, whereas an increase of 100 mg L^−1^ is acceptable for streams^22^. Similarly, hazard assessment of MP in different systems would eventually require that effect-thresholds are established for the critical MP concentration, such as NOE%MP, relative to the background TSS levels and their combined interactions with biota.

By using reference particles – natural minerals or standardised plastics – with predictable effects on the test organisms, one may identify and account for the general responses anticipated from exposure to suspended solids. The relative importance of MP addition to the suspension could thereby be assessed. The hazard level we found for MP contribution to SS using a planktonic organism is close to the EC_10_ of 1.1% MP per sediment dry weight, reported for a benthic macroinvertebrate^38^. This could further support the possibility to benchmark MP effects against the lower 95%-confidence bound of the reference material and use the corresponding contribution of the MP to the mixture as the environmentally safe MP level (Fig. 4).

The hazard assessment and the regulatory framework for MP contaminants in aquatic systems require integration with an assessment of particulate matter pollution at large because the approaches required to establish safe thresholds are similar. For example, raising levels of black carbon in the atmosphere implies increased inputs of these particles in the aquatic systems, where their environmental effects are also a matter of concern^58^. Addressing all types of particulate pollution and focusing on physicochemical properties of these particles would provide a translational value when developing testing and regulatory practices.

## Supplementary information

Supplementary Information

Dataset 1
