# Supplementary material for: A novel method for assessing microplastic effect in suspension through mixing test and reference materials
Source: Sci Rep. 2019 Jul 23;9:10695. doi: 10.1038/s41598-019-47160-1 (PMC6650601; doi:10.1038/s41598-019-47160-1)
Supplement: Supplementary file 1 — Supplementary Information [file 41598_2019_47160_MOESM1_ESM.docx]

**Supplementary Information**

Zandra Gerdes, Markus Hermann, Martin Ogonowski and Elena Gorokhova*

Department of Environmental Science and Analytical Chemistry, Stockholm University, Svante Arrhenius väg 8, SE-11418 Stockholm, Sweden.

**A novel method for assessing microplastic effect in suspension through mixing test and reference materials**

9 pages, 2 tables, 5 figures

**Table S1.** Mass- (mg/L) and particle-based (number of particles per liter, #/L) concentrations of PET and kaolin in the experimental suspensions with varying percentage of PET (%MP). The particle-based concentrations were calculated using the size spectra of PET and kaolin (Fig. S3) and a specific gravity of each material (PET: 1.4 g/cm^3^; kaolin: 2.6 g/cm^3^). Concentrations depicted in Italics were not used in the dose-response analysis (Fig. 3).

| Mass-based concentration, mg/L | %MP | PET, #/L | Kaolin, #/L | Total, #/L |
| --- | --- | --- | --- | --- |
| *0.1* | *0* | *0* | *359* | *359* |
| *0.1* | *100* | *290* | *0* | *290* |
| *1* | *0* | *0* | *3594* | *3594* |
| *1* | *100* | *2901* | *0* | *2901* |
| 10 | 0 | 0 | 35942 | 35942 |
| 10 | 20 | 5801 | 28754 | 34555 |
| 10 | 40 | 11603 | 21565 | 33168 |
| 10 | 60 | 17404 | 14377 | 31781 |
| 10 | 80 | 23206 | 7188 | 30394 |
| 10 | 100 | 29007 | 0 | 29007 |
| 100 | 0 | 0 | 359424 | 359424 |
| 100 | 20 | 58014 | 287539 | 345553 |
| 100 | 40 | 116028 | 215654 | 331682 |
| 100 | 60 | 174042 | 143769 | 317812 |
| 100 | 80 | 232057 | 71885 | 303941 |
| 100 | 100 | 290071 | 0 | 290071 |
| 1000 | 0 | 0 | 3594235 | 3594235 |
| 1000 | 20 | 580142 | 2875388 | 3455530 |
| 1000 | 40 | 1160283 | 2156541 | 3316824 |
| 1000 | 60 | 1740425 | 1437694 | 3178119 |
| 1000 | 80 | 2320566 | 718847 | 3039413 |
| 1000 | 100 | 2900708 | 0 | 2900708 |
| 10000 | 0 | 0 | 35942355 | 35942355 |
| 10000 | 20 | 5801424 | 28753876 | 34555300 |
| 10000 | 40 | 11602832 | 21565407 | 33168240 |
| 10000 | 60 | 17404251 | 14376939 | 31781190 |
| 10000 | 80 | 23205662 | 7188468 | 30394130 |
| 10000 | 100 | 29007080 | 0 | 29007080 |

**Table S2.** Mortality of *Daphnia magna* in the dose-response experiments with PET-kaolin mixtures; *n* is the number of runs (all data). See SI2.xls (Supporting Information) for the data used in the dose-response analysis (Fig. 3).

| Treatment | | Proportion of dead individuals | | |
| --- | --- | --- | --- | --- |
| %MP | SS, mg/L | mean | SD | *n* |
| 0 | 0 | 0.05 | 0.06 | 43 |
|  | *0.1* | *0.00* | *0.00* | *5* |
|  | *1* | *0.00* | *0.00* | *5* |
|  | 10 | 0.04 | 0.07 | 19 |
|  | 100 | 0.24 | 0.23 | 20 |
|  | 1000 | 0.72 | 0.24 | 24 |
|  | 10000 | 1.00 | 0.00 | 25 |
| 20 | 0 | 0.00 | 0.00 | 3 |
|  | 10 | 0.12 | 0.04 | 5 |
|  | 100 | 0.65 | 0.13 | 4 |
|  | 1000 | 0.82 | 0.13 | 5 |
|  | 10000 | 0.96 | 0.06 | 5 |
| 40 | 0 | 0.10 | 0.00 | 3 |
|  | 10 | 0.08 | 0.10 | 4 |
|  | 100 | 0.76 | 0.05 | 5 |
|  | 1000 | 0.52 | 0.11 | 5 |
|  | 10000 | 0.92 | 0.08 | 5 |
| 60 | 0 | 0.10 | 0.00 | 4 |
|  | 10 | 0.24 | 0.09 | 5 |
|  | 100 | 0.62 | 0.13 | 5 |
|  | 1000 | 0.56 | 0.05 | 5 |
|  | 10000 | 0.94 | 0.06 | 5 |
| 80 | 0 | 0.10 | 0.00 | 4 |
|  | 10 | 0.24 | 0.11 | 5 |
|  | 100 | 0.60 | 0.14 | 5 |
|  | 1000 | 0.42 | 0.04 | 5 |
|  | 10000 | 0.92 | 0.08 | 5 |
| 100 | 0 | 0.05 | 0.08 | 5 |
|  | 1 | 0.07 | 0.12 | 3 |
|  | 10 | 0.16 | 0.05 | 5 |
|  | 100 | 0.68 | 0.12 | 6 |
|  | 1000 | 0.78 | 0.08 | 6 |
|  | 10000 | 0.98 | 0.04 | 6 |

**Text 1. Polymer characterization by Fourier Transform Infrared (FTIR) Spectroscopy**

PET purchased from Goodfellow GmbH, product number ES306312, was analyzed using attenuated total reflection Fourier transform infrared spectroscopy (ATR-FTIR). ATR-FTIR spectra were obtained using a Bruker Vertex 70 FTIR spectrometer equipped with a platinum ATR crystal. Scans were performed from 4000 cm^-1^ to 400 cm^-1^. The trace is the average of two scans with a 4 cm^-1^ resolution. Spectra were generated using OPUS Spectroscopy software. PET was identified by cross-reference with Jung et al. (2018) using focusing on the absorption bands described therein, which are typical of the polymers identification (depicted in Fig. S5).^1,2^


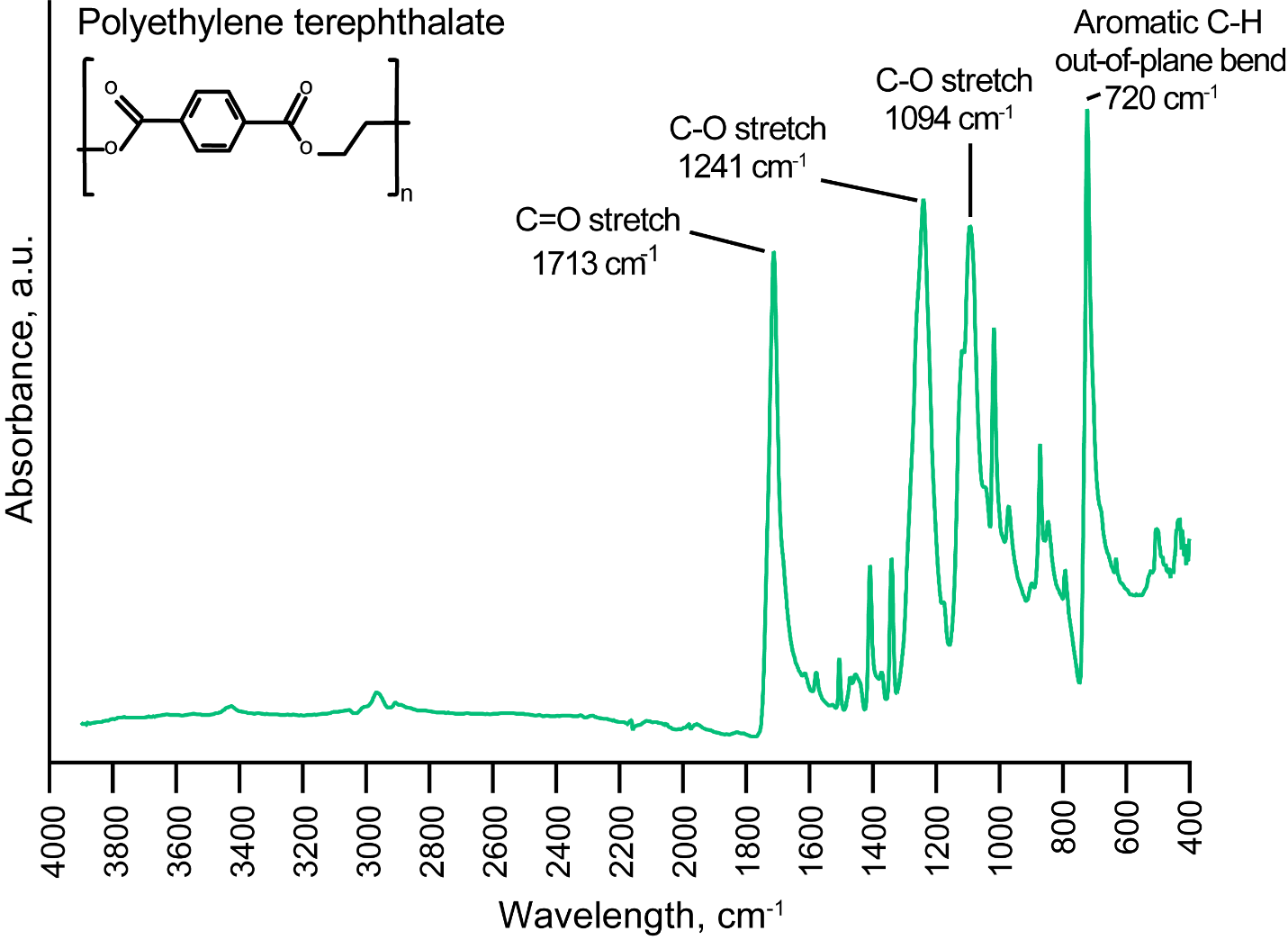


**Figure S1.** The absorbance spectra of the PET polymer used in this study. Spectral range 4000-400 cm^-1^.

**Text 2. Particle preparation and size distributions**

The PET powder mixed with milliQ water containing 0.01 % v/v of a non-ionic surfactant (Tween 80, Sigma-Aldrich) was sequentially wet-sieved (200-, 100- and 40-µm sieves) to produce a size fraction as similar to the kaolin particle size range as possible. The <40 µm particles were vacuum filtered through a metal Büchner funnel fitted with a 0.2-µm nylon membrane (Merck Millipore, GNWP04700). The filtrate was dried, weighed and re-suspended for size distribution analysis. The concentration of Tween-80 in the experimental system never exceeded 0.0001%, which is well below the levels regarded as non-toxic by OECD guidelines^3^

The size distribution of the particles was measured with a Spectrex laser particle counter (Spectrex, model PC-2000, Redwood City, USA). The instrument utilizes the near-angle light scattering principle by passing a rotating laser beam through the glass container and then focusing the near-angle light pulses, resulting from the laser beam/particle collisions, on to the photodetector for conversion into particle count and size data. Small vial attachment was used to determine the size and number of particles in the 3-100 µm range (integrated mode, filters F11 and F81); however, detection of particles in 2 to 3 µm range was less precise. The performance was verified daily by measuring a standard of known particle size and counts. The standard, provided by the instrument manufacturer, contained polystyrene spheres (4.2 µm in diameter) in an alcohol/freon liquid matrix. Every effort was made to minimize background noise on these measurements such as cleaning the vial exterior to remove surface contaminants. Appropriate blanks (standard blank sample provided by the manufacturer and the particle-free water used to dilute the samples) and controls (reference sample provided with the instrument and kaolin sample with known TSS concentration) were used. Testing replicate samples of the polymer and kaolin standards showed that the variation of particle counts is less than 6 %.

Care was taken to homogenize suspensions by sonicating the samples before the measurements for five seconds and use a magnet stirrer during the measurements to prevent sedimentation. Both PET and kaolin particles showed a unimodal size distribution with the highest fraction of particles ≤3 µm in diameter. Few particles of either material were larger than eleven µm, and ~90 % were between three and ten µm (Fig. S5). As particle size distributions were not normally distributed, they were represented with median and interquartile range (Fig. S6)

**Figure S2.** The relative size distribution and a cumulative fraction of PET and kaolin particles measured with a laser particle counter in deionized water with 0.01 % Tween-80.


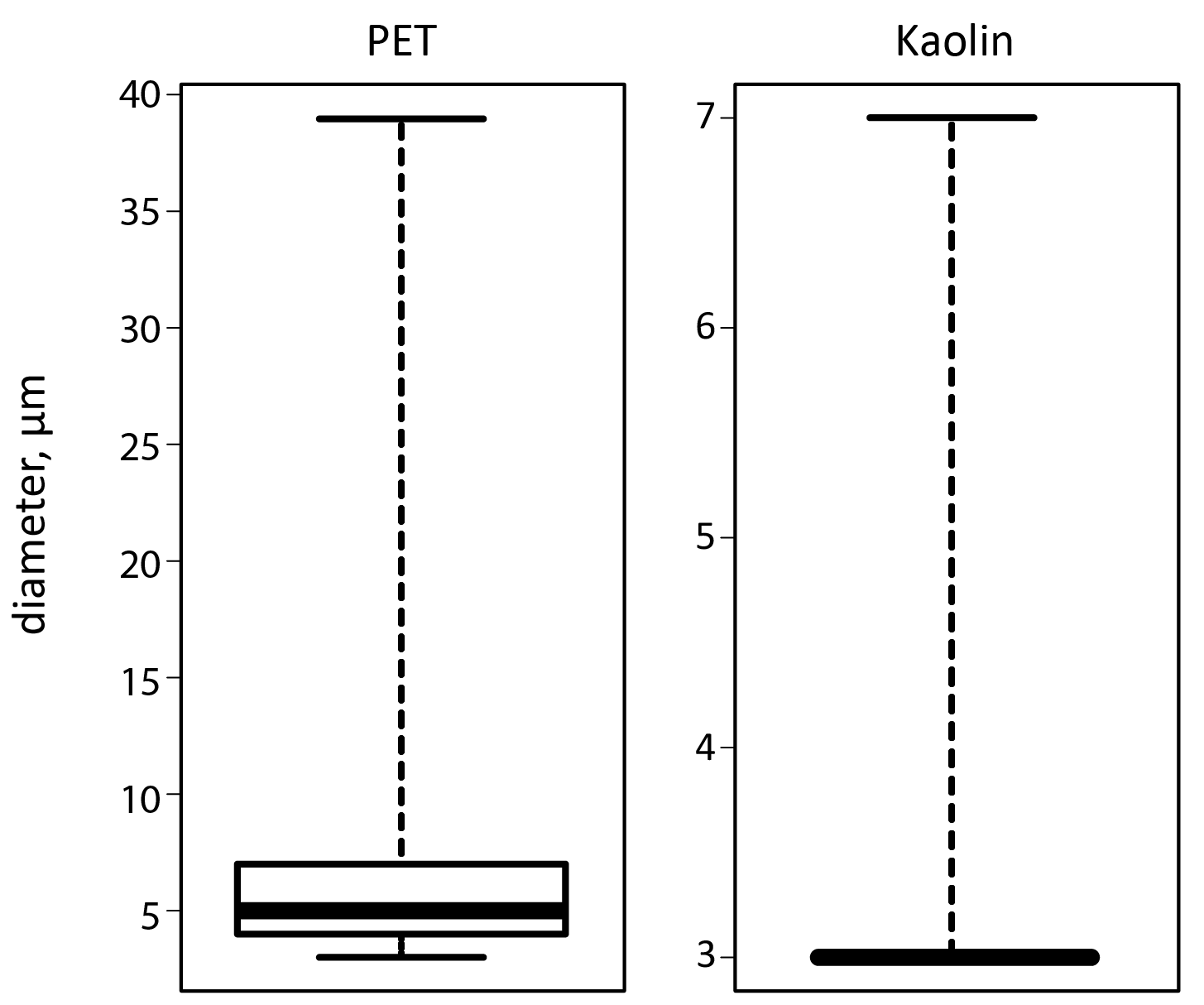


**Figure S3**. The size distribution of PET and kaolin particles based on measurements with a laser particle counter Spectrex 2000 (2-100 μm measurement range). The boxplots show the median as the thick line, 25^th^ and 75^th^ percentiles as the box and whiskers.

**B**

**A**

**Figure S4.** Light-microscopy photograph of PET (A) and kaolin (B) dispersed by sonication and used for preparing test mixtures.

A

B


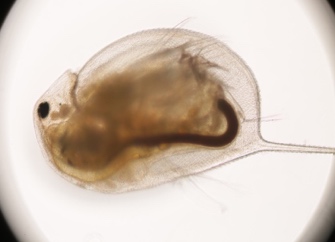


**Figure S5.** Experimental system and test animals. (A): The experimental units (50-mL test tubes) were mounted on a plankton wheel rotating at 0.5 rpm and turning the tubes upside down. The rotation speed was selected to ensure no visible sedimentation and thus constant encounter rate of the particles during the rotation cycle. (B): As test organism, we used *Daphnia magna* that was observed to ingest particles in the test size range; this individual was photographed after a one-hour exposure to kaolin visible as the dark matter in the gut. Continuous particle ingestion by the test animals was confirmed for both materials through observation of the exposed animals by bright-field microscopy.

**References**

(1) Jung, M. R.; Horgen, F. D.; Orski, S. V.; Rodriguez C., V.; Beers, K. L.; Balazs, G. H.; Jones, T. T.; Work, T. M.; Brignac, K. C.; Royer, S.-J.; et al. Validation of ATR FT-IR to Identify Polymers of Plastic Marine Debris, Including Those Ingested by Marine Organisms. *Marine Pollution Bulletin* **2018**, *127*, 704–716.

(2) Chércoles Asensio, R.; San Andrés Moya, M.; de la Roja, J. M.; Gómez, M. Analytical Characterization of Polymers Used in Conservation and Restoration by ATR-FTIR Spectroscopy. *Anal Bioanal Chem* **2009**, *395* (7), 2081–2096.

(3) OECD. Organization for Economic Cooperation and Development. Guideline for the Testing of Chemicals 218: Sediment-Water Chironomid Toxicity Test Using Spiked Sediment. Adopted 13 April 2004. Paris, France. 2004.
